# Improving antenatal detection of small‐for‐gestational‐age fetus: economic evaluation of Growth Assessment Protocol

**DOI:** 10.1002/uog.26022

**Published:** 2022-11-01

**Authors:** S. Relph, M. C. Vieira, A. Copas, K. Coxon, A. Alagna, A. Briley, M. Johnson, L. Page, D. Peebles, A. Shennan, B. Thilaganathan, N. Marlow, C. Lees, D. A. Lawlor, A. Khalil, J. Sandall, D. Pasupathy, A. Healey, on behalf of the DESiGN Trial Team

**Affiliations:** ^1^ Department of Women and Children's Health, School of Life Course Sciences, Faculty of Life Sciences and Medicine King's College London London UK; ^2^ Department of Obstetrics and Gynaecology University of Campinas (UNICAMP), School of Medical Sciences São Paulo Brazil; ^3^ Centre for Pragmatic Global Health Trials Institute for Global Health, University College London London UK; ^4^ Faculty of Health, Social Care and Education Kingston and St George's University London UK; ^5^ The Guy's & St Thomas' Charity London UK; ^6^ Caring Futures Institute College of Nursing and Health Sciences, Flinders University Adelaide Australia; ^7^ Department of Surgery and Cancer Imperial College London London UK; ^8^ West Middlesex University Hospital, Chelsea & Westminster Hospital NHS Foundation Trust London UK; ^9^ UCL Institute for Women's Health University College London London UK; ^10^ Fetal Medicine Unit St George's University Hospitals NHS Foundation Trust London UK; ^11^ Molecular & Clinical Sciences Research Institute St George's, University of London London UK; ^12^ Population Health Science Bristol Medical School, University of Bristol Bristol UK; ^13^ Bristol NIHR Biomedical Research Centre Bristol UK; ^14^ Reproduction and Perinatal Centre, Faculty of Medicine and Health University of Sydney Sydney Australia; ^15^ Department of Health Service and Population Research David Goldberg Centre, King's College London London UK

**Keywords:** antenatal screening, cost‐effectiveness, economic evaluation, growth assessment protocol, SGA

## Abstract

**Objective:**

To determine whether the Growth Assessment Protocol (GAP), as implemented in the DESiGN trial, is cost‐effective in terms of antenatal detection of small‐for‐gestational‐age (SGA) neonate, when compared with standard care.

**Methods:**

This was an incremental cost‐effectiveness analysis undertaken from the perspective of a UK National Health Service hospital provider. Thirteen maternity units from England, UK, were recruited to the DESiGN (DEtection of Small for GestatioNal age fetus) trial, a cluster randomized controlled trial. Singleton, non‐anomalous pregnancies which delivered after 24 + 0 gestational weeks between November 2015 and February 2019 were analyzed. Probabilistic decision modeling using clinical trial data was undertaken. The main outcomes of the study were the expected incremental cost, the additional number of SGA neonates identified antenatally and the incremental cost‐effectiveness ratio (ICER) (cost per additional SGA neonate identified) of implementing GAP. Secondary analysis focused on the ICER per infant quality‐adjusted life year (QALY) gained.

**Results:**

The expected incremental cost (including hospital care and implementation costs) of GAP over standard care was £34 559 per 1000 births, with a 68% probability that implementation of GAP would be associated with increased costs to sustain program delivery. GAP identified an additional 1.77 SGA neonates per 1000 births (55% probability of it being more clinically effective). The ICER for GAP was £19 525 per additional SGA neonate identified, with a 44% probability that GAP would both increase cost and identify more SGA neonates compared with standard care. The probability of GAP being the dominant clinical strategy was low (11%). The expected incremental cost per infant QALY gained ranged from £68 242 to £545 940, depending on assumptions regarding the QALY value of detection of SGA.

**Conclusion:**

The economic case for replacing standard care with GAP is weak based on the analysis reported in our study. However, this conclusion should be viewed taking into account that cost‐effectiveness analyses are always limited by the assumptions made. © 2022 The Authors. Ultrasound in Obstetrics & Gynecology published by John Wiley & Sons Ltd on behalf of International Society of Ultrasound in Obstetrics and Gynecology.

**CONTRIBUTION**

**What are the novel findings of this work?**

The Growth Assessment Protocol (GAP), as implemented in the DESiGN (DEtection of Small for GestatioNal age fetus) trial, is expected to cost an additional £34 559 per 1000 births. In terms of cost‐effectiveness, it is most likely that GAP is both more clinically effective and more costly than standard care (44% probability), while the chances of it being both more clinically effective and less costly are low.

**What are the clinical implications of this work?**

When implemented as it was in sites recruited to the DESiGN trial, the economic case for replacing standard care with GAP to improve antenatal detection of small‐for‐gestational age and stillbirth is weak.

## INTRODUCTION

Reducing the prevalence of stillbirth is a global priority^1^. In high‐income countries, approximately four in every 10 stillborn babies are growth‐restricted^2^. Therefore, stillbirth prevention strategies target risk assessment, antenatal diagnosis, surveillance and timely birth of small‐for‐gestational‐age (SGA) babies (fetal/birth weight < 10^th^ centile)^3^, ^4^. The Growth Assessment Protocol (GAP) is a complex intervention that aims to prevent stillbirth by improving antenatal care and detection of SGA.

The DESiGN (DEtection of Small for GestatioNal age fetus) trial was the first pragmatic cluster randomized controlled trial that compared the effect of GAP against standard care in the UK^5^, finding no statistically significant difference in the rate of ultrasound detection of SGA (primary outcome) (25.9% *vs* 27.7%; adjusted difference, 2.4% (95% CI, –6.1 to 10.8%); *P* = 0.58)^6^.

Economic evaluation in healthcare research is recommended to assist decision‐making regarding the adoption and extent of implementation of an intervention^7^. An economic evaluation studying the cost‐effectiveness of GAP has not yet been published. The objective of this study was to determine whether GAP is a cost‐effective approach to improving antenatal detection of SGA and preventing stillbirth within hospitals implementing the GAP program, compared with hospitals following standard practice.

## METHODS

This report was written in accordance with the Consolidated Health Economic Evaluation Reporting Standards (CHEERS)^8^. The trial was registered with the ISRCTN registry (ISRCTN67698474). An analysis plan (available on request) was developed in 2019 and approved by the joint Steering and Data Monitoring Committee. Further details of the methods are provided in Appendix S1.

### Study design

A probabilistic cost‐effectiveness analysis was undertaken using decision‐analytic methods applied to clinical data from the DESiGN trial. Costs were estimated from the perspective of a National Health Service (NHS) provider. Costs and clinical outcomes in hospitals randomized to implement GAP were compared with those in hospitals randomized to continue standard care. Ethical approval for the DESiGN trial was obtained through the Health Research Authority Integrated Research Applications System from the London Bloomsbury Research Ethics Committee (Ref. 15/LO/1632) and the Confidentiality Advisory Group (Ref. 15/CAG/0195).

### Trial design, population and setting

Thirteen cluster sites (English maternity units/hospitals, predominantly in London) were allocated randomly to the GAP intervention (*n* = 7) or to standard care (*n* = 6) between November 2015 and July 2017. Two cluster sites allocated to GAP withdrew before commencing implementation due to concerns over its expected financial impact. Neither site was therefore used to evaluate the effect of the intervention, although their data were used for other purposes, as explained later. The five remaining cluster sites implemented GAP^6^.

All women delivering in a cluster site were included in the trial database. Cases with a multiple birth, congenital fetal anomaly or birth before 24 + 1 weeks were excluded from the analysis. Data were collected for births during the trial outcome comparison phase (1 September 2018 to 28 February 2019 for most sites) and during the prerandomization phase (1 year prior to cluster randomization) for baseline adjustments.

### Intervention and standard care

The GAP intervention was designed by a team at The Perinatal Institute, Birmingham, UK. It involves additional staff training, stratification of pregnant women according to risk of SGA, SGA screening protocols that differ according to risk strata, use of fetal or birth‐weight centiles customized to the woman's (height, weight, parity, ethnicity) and the baby's (sex and gestational age) characteristics to define SGA, and audit of missed cases of SGA^9^. Standard care was defined as the screening strategy already implemented within the allocated clusters, influenced by the Royal College of Obstetricians and Gynaecologists guideline^10^. The trial protocol specified that these sites should not implement GAP or assess fetal or neonatal size using customized growth charts.

### Time horizon

Fetal surveillance according to GAP commenced at 24 weeks' gestation. Costs incurred before this gestational age were not expected to vary according to study arm and were not included. After 24 weeks, we included all major antenatal, intrapartum, neonatal and postnatal costs until the mother or infant was discharged from the care episode that included birth. Costs were not discounted, because all were expected to occur within a single year.

### Trial data for economic evaluation

All data required for analysis of clinical outcomes and costs were obtained from routine electronic patient records (EPRs). Our data collection and management methods, including a detailed description of data quality checks, have been published previously^11^.

### Clinical outcome

The primary outcome of the DESiGN trial was antenatal detection of SGA (positive ultrasound screening, as defined by estimated fetal weight < 10^th^ centile at the last fetal growth scan, using Hadlock centiles in the standard care arm and Gestation‐Related Optimal Weight (GROW) centiles in the intervention arm)^12^, ^13^ in a fetus confirmed to be SGA at birth (defined as birth weight < 10^th^ centile by both UK90 population reference charts and GROW charts)^13^, ^14^. In this economic analysis, screening outcomes for both SGA (true positive and false negative) and non‐SGA (false positive and true negative) babies were studied. For the sensitivity analysis, we also present results based on a secondary definition of SGA used in the clinical trial and one that is more likely to be used in routine practice. One GAP‐implementing site was excluded from the analysis of false‐positive and true‐negative cases because it did not provide data enabling us to derive these.

### Resource utilization

Data for all significant antenatal, intrapartum, postnatal and neonatal activities were collected from the EPRs (Appendix S2). Costs were calculated by multiplying units of activity by the unit cost. The costs were then summed to obtain antenatal, intrapartum, postnatal and neonatal subtotals and a total cost for each birth was calculated.

For estimating resource use during the antenatal period, data from ultrasound scans and antenatal inpatient admissions were generally available. Data from antenatal appointments and unscheduled outpatient attendances were missing either completely or partially (but systematically) for five of the six standard‐care sites. To maximize the number of clusters available for antenatal cost analysis, these resource items were excluded from the base‐case analysis. This pragmatic decision was guided by the hypothesis that the main cost impact of GAP would most likely arise from an increased number of scans. The effect of excluding these data was explored further by sensitivity analysis. The primary economic analysis was subsequently carried out using nine cluster sites for antenatal costs (standard care, *n* = 5; intervention, *n* = 4), as one site in each trial arm did not contribute data on antenatal hospital admissions. Unlike antenatal care costs, subsequent costs were expected to vary according to screening outcome but not treatment arm. They were, therefore, included without stratification according to treatment arm, and all 13 sites contributed data to calculate intrapartum costs. Due to the limited availability of data, only nine sites were used to calculate postnatal costs (standard care, *n* = 3; intervention, *n* = 6) and 11 sites were used to calculate neonatal costs (standard care, *n* = 4; intervention, *n* = 7).

Data on activities relevant to GAP implementation were also collected. Information on the number and type of staff members employed was collected from the clinical leads at each site. The number of staff members from each professional group (doctors, midwives and sonographers) who attended the site‐wide training launches was obtained from the intervention provider. The time taken to complete each training type was estimated as the median time reported by participants in semi‐structured interviews conducted during the trial process evaluation^5^. The fee charged by the GAP provider, including a one‐off set‐up cost of £500 and an annual cost that differed according to the expected annual birth rate at each site, was also included (Appendix S3). No evidence was found during interviews that the generation or use of GAP fetal growth charts had changed the expected antenatal clinic appointment duration (midwives or sonographers were still expected to see the same number of women during a session, even if this incurred a loss of rest breaks). Therefore, the costs for these activities were not included.

### Unit costs

Unit costs for each maternity or neonatal care activity were estimated following a systematic review of maternity costs published as part of economic evaluations conducted in the UK^15^ and a review of the available costs published by the Department of Health as part of the national maternity tariff from 2015 to 2016 and 2017 to 2018 (Appendix S3)^16^, ^17^. Costs were then inflated, when appropriate, to 2018 to 2019 prices^18^, ^19^. Hourly costs were estimated for each staff group using Unit Costs of Health and Social Care 2018 data published by the Personal Social Services Research Unit (Appendix S3)^20^.

### Modeling approach

**Figure 1:**
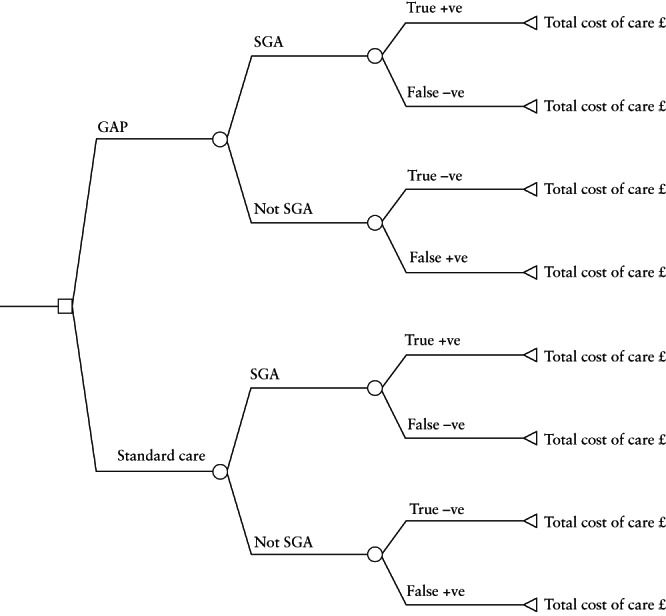
Decision‐analytic model linking costs associated with standard care *vs* Growth Assessment Protocol (GAP) and the four mutually exclusive screening outcomes. +ve, positive; –ve, negative; SGA, small‐for‐gestational age.

The cost‐effectiveness model linked the costs of care to the four mutually exclusive screening outcomes (Figure 1). The two main modeling outputs were: the total cost of hospital care per 1000 births using GAP or standard care (sum of antenatal, intrapartum, postnatal and neonatal care costs with/without GAP implementation costs) and the number of true‐positive screening outcomes per 1000 births expected under GAP or standard care. These outputs were then used to evaluate the probability of four possible conclusions regarding the data: (1) GAP is associated with lower cost of care and more true‐positive cases of SGA (GAP is the ‘dominant’ clinical strategy); (2) GAP is associated with higher cost and more true‐positive SGA cases (a trade‐off); (3) GAP is associated with higher cost and fewer true‐positive SGA cases (standard care is ‘dominant’); or (4) GAP is associated with lower cost and fewer true‐positive SGA cases (a trade‐off). If the second conclusion held true, we planned to estimate the incremental cost‐effectiveness ratio (ICER) for GAP, i.e. the expected incremental cost per additional true‐positive SGA neonate identified.

To reflect uncertainty in the input parameters, expected principally from trial sampling error, the cost‐effectiveness analysis was conducted probabilistically. Uncertainty around intervention cost‐effectiveness was therefore presented as the probability of observing alternative cost‐effectiveness outcomes and by plotting a 95% confidence ellipse for the cost‐effectiveness plane^21^.

### Estimation of input parameters

For the main trial, a cluster‐summary approach was used to extract statistical information required for probabilistic economic modeling^22^. Multivariate analysis of individual screening outcomes and costs, adjusted for maternal age, parity, ethnicity and body mass index (the latter for cost outcomes only), was conducted to obtain cluster‐level predicted values for the proportion of births and the mean cost per birth (for each subtotal of hospital care) associated with each screening outcome during the trial outcome comparison phase. Cluster summary values for the proportion of births expected to be SGA and non‐SGA were based on the unadjusted mean value for site clusters.

For input parameters that are potentially subject to a treatment effect of GAP (proportion of SGA or non‐SGA births in which SGA was detected antenatally and antenatal costs according to screening outcome), the two sites allocated to GAP that did not attempt implementation (as per the main trial analysis^5^) were excluded. Linear regression models were fitted to cluster summary values and used to generate probability distributions for each treatment allocation; for screening outcomes expressed as cluster‐level proportions, a linear regression model was fitted to the logit transformation of the observed outcome, with a retransformation to obtain the predicted proportion in order to avoid deriving predictions with implausible values. Linear predictions according to treatment allocation were also adjusted for trial baseline outcomes (derived from births during the pre‐implementation phase) for each site cluster and a trial stratification variable. Monte‐Carlo simulation was used to obtain 10 000 random draws from a multivariate normal distribution of linear predictions according to treatment allocation with adjustments made to predictions using the Cholesky decomposition method to account for correlation between regression parameters^23^.

For the remaining input parameters (proportion of SGA and non‐SGA neonates and intrapartum, postnatal and neonatal costs according to screening outcome), probability distributions were again generated using Monte‐Carlo simulation from a prespecified probability distribution. Probability distributions were parameterized using the relevant cluster summary data. In all cases, the selection of an appropriate distribution was guided by the need to generate a plausible range of parameter values (i.e. cost per birth constrained to be ≥ 0 and SGA/non‐SGA proportions bounded by the values 0 and 1).

### Sensitivity analysis

To assess whether exclusion of unscheduled and scheduled outpatient attendances or appointments from antenatal costs may have introduced bias into comparisons between GAP and standard care, our probabilistic analysis of the total cost of hospital care was repeated for two alternative scenarios: the base‐case plus an uplift for unscheduled attendance costs and the base‐case plus an uplift for scheduled clinic appointment costs (uplifts calculated from utilization of the resource type in clusters that provided quality data on these). The sensitivity of our main conclusions to the use of an alternative definition of SGA status at birth was also tested. The secondary definition of SGA was birth weight < 10^th^ percentile according to population charts for standard care and according to the customized standard (GROW charts) for GAP^12^, ^13^.

### Secondary analysis

To aid interpretation of our findings, a secondary analysis was performed to recalibrate the antenatal detection of SGA births into neonate quality‐adjusted life year (QALY) gains arising from prevention of stillbirth. QALYs are the accepted outcome metric for establishing whether new healthcare technologies are a cost‐effective use of NHS resources^24^. These values were combined with our existing model to determine whether the incremental cost and QALY implications associated with GAP satisfied the cost‐effectiveness criteria currently used by the UK National Institute for Health and Care Excellence (NICE) to guide NHS resource allocation. NICE criteria stipulate that the cost of new health technologies should not exceed £20 000 to £30 000 for every QALY gained^24^.

Clinical trial data were used to determine the baseline incidence of stillbirth among SGA births in which SGA was not detected antenatally. Previous studies have suggested that 50% of stillbirths occurring among cases with undetected SGA could be avoided if SGA were detected^25^, ^26^, ^27^. NICE estimate that prevention of one stillbirth gains 23.73 (range, 15–30) discounted QALYs (applying a discount rate of 3.5%). Combining this evidence, three estimates of the QALY benefit per SGA birth detected antenatally were derived and applied in our secondary analysis: a ‘central’ estimate (assuming that 50% of stillbirths linked to undetected SGA are prevented with 23.73 QALYs gained per stillbirth avoided); a ‘high’ (and highly optimistic) estimate (all stillbirths prevented, 30 QALYs gained); and a ‘low’ estimate (25% of stillbirths prevented, 15 QALYs gained).

As an extension of our secondary analysis, a conditional incremental net benefit (INB) analysis was also performed^28^. This was used to assess whether a cost‐effective rate of antenatal detection of SGA under GAP is likely to be achievable given a plausible distribution of values for this parameter extracted from the trial data. The analysis was repeated under the varying assumptions regarding the QALY value of detecting a SGA birth antenatally, as described above. To implement the conditional INB analysis, the QALY benefit of early detection was monetized and assessed according to the NICE cost‐effectiveness threshold. Subtracting incremental monetized benefits from the incremental costs gave the INB of GAP. If INB > 0, then GAP was considered cost‐effective at the chosen threshold level, which was the lower value of £20 000 per QALY gained, preferred by NICE. The INB was estimated at varying levels of the SGA detection rate corresponding to the deciles within the distribution for this parameter. All other parameters were varied probabilistically (as described earlier).

## RESULTS

The economic modeling drew on data from 209 314 pregnancies, of which 19 312 pregnancies (nine sites) were analyzed for antenatal costs during the comparison phase, 85 575 pregnancies (13 sites) were analyzed for intrapartum costs, 60 071 pregnancies (nine sites) were analyzed for postnatal costs and 73 006 pregnancies (11 sites) were analyzed for neonatal costs. For clinical outcomes during the comparison phase, 13 810 pregnancies in the standard‐care arm and 8882 pregnancies in the intervention arm were analyzed. For adjustments of clinical outcomes using baseline data, 29 404 pregnancies were included in the standard‐care arm and 21 596 pregnancies were included in the intervention arm. The consort diagram, characteristics of women included during the prerandomization and comparison phases and results of the analysis for primary and secondary clinical outcomes have been published previously^6^.

Model parameter values estimated from the trial data based on the primary and secondary definitions of small baby identified at birth are described in Table 1. The expected total cost of all hospital care per 1000 births was estimated to be £23 763 higher using GAP compared with standard care, with a 62% probability that GAP would increase hospital care costs. The cost of implementing GAP (staff training costs and license fees) was estimated to be an additional £10 796 per 1000 births. The total expected incremental cost of GAP compared with standard care was £34 559 more per 1000 births, with a 68% probability that GAP would be costlier compared with standard care (Tables 2 and 3).

**Table 1:** Model input parameters according to whether primary or secondary definition of small‐for‐gestational‐age (SGA) neonate was used (*n* = 10 000 model simulations)

Model input parameter	Primary SGA definition		Secondary SGA definition	
	Value	Probability distribution	Value	Probability distribution
Clinical outcome				
Under standard care		Multivariate normal		Multivariate normal
% SGA neonates TP of all SGA neonates	28.5 (20.0–35.8)	—	28.3 (20.2–35.1)	—
% non‐SGA neonates FP of all non‐SGA neonates	1.6 (1.0–2.0)	—	1.6 (1.0–2.0)	—
Under GAP		Multivariate normal		Multivariate normal
% SGA neonates TP of all SGA neonates	30.9 (21.3–39.1)	—	30.8 (21.5–38.6)	—
% non‐SGA neonates FP of all non‐SGA neonates	2.3 (1.3–2.9)	—	2.4 (1.4–3.0)	—
Antenatal care cost				
Cost incurred with each screening outcome under standard care (per birth)				
TP	£1276 (£1098 to £1438)	Gamma: α = 25.5, β = 50.1	£1263 (£1087 to £1416)	Gamma: α = 28.0, β = 45.1
FN	£848 (£767 to £923)	Gamma: α = 53.3, β = 15.9	£829 (£752 to £903)	Gamma: α = 55.2, β = 15.0
TN	£670 (£614 to £759)	Gamma: α = 42.0, β = 16.4	£690 (£617 to £758)	Gamma: α = 42.2, β = 16.3
FP	£1074 (£900 to £1224)	Gamma: α = 19.4, β = 55.2	£1075 (£887 to £1236)	Gamma: α = 16.8, β = 64.1
Cost incurred with each screening outcome under GAP (per birth)		NA†		NA†
TP	£1508 (£1231 to £1781)	—	£1428 (£1176 to £1677)	—
FN	£894 (£811 to £970)	—	£826 (£743 to £905)	—
TN	£689 (£606 to £766)	—	£688 (£606 to £764)	—
FP	£846 (£608 to £1069)	—	£750 (£511 to £966)	—
Incremental effect of GAP on antenatal cost		Multivariate normal		Multivariate normal
TP	£232 (£13 to £451) [–£406 to £861]	—	£164 (–£24 to £356) [–£387 to £718]	—
FN	£45 (£33 to £58) [£8 to £82]	—	–£4 (–£33 to £25) [–£89 to £79]	—
TN	–£1 (–£38 to £35) [–£109 to £107]	—	–£2 (–£39 to £35) [–£107 to £108]	—
FP	–£233 (–£402 to –£66) [–£711 to £248]	—	–£336 (–£492 to –£181) [–£780 to £122]	—
Cost incurred with each screening outcome from intrapartum care (per birth)*				
TP	£3022 (£2964 to £3079)	Gamma: α = 1237.2, β = 2.4	£2996 (£2938 to £3053)	Gamma: α = 1202.6, β = 2.5
FN	£2724 (£2672 to £2776)	Gamma: α = 1237.2, β = 2.2	£2699 (£2645 to £2751)	Gamma: α = 1202.6, β = 2.2
TN	£2708 (£2665 to £2752)	Gamma: α = 1744.6, β = 1.6	£2711 (£2666 to £2754)	Gamma: α = 1707.6, β = 1.6
FP	£2801 (£2756 to £2847)	Gamma: α = 1744.5, β = 1.6	£2791 (£2746 to £2836)	Gamma: α = 1707.6, β = 1.6
Costs incurred with each screening outcome from postnatal care (per birth)*				
TP	£729 (£552 to £875)	Gamma: α = 8.7, β = 83.8	£693 (£536 to £824)	Gamma: α = 9.7, β = 70.9
FN	£467 (£354 to £562)	Gamma: α = 8.7, β = 53.4	£451 (£348 to £538)	Gamma: α = 9.8, β = 46.0
TN	£364 (£293 to £426)	Gamma: α = 13.3, β = 27.1	£357 (£286 to £419)	Gamma: α = 12.7, β = 28.2
FP	£561 (£452 to £655)	Gamma: α = 13.3, β = 42.0	£547 (£438 to £642)	Gamma: α = 12.7, β = 43.0
Costs incurred with each screening outcome from neonatal care (per birth)*				
TP	£2803 (£1859 to £3524)	Gamma: α = 4.7, β = 593.6	£2767 (£1916 to £3453)	Gamma: α = 5.5, β = 509.8
FN	£1110 (£670 to £1269)	Gamma: α = 4.7, β = 212.1	£1177 (£707 to £1512)	Gamma: α = 3.5, β = 341.2
TN	£416 (£305 to £508)	Gamma: α = 7.4, β = 56.4	£405 (£287 to £502)	Gamma: α = 6.3, β = 64.6
FP	£2351 (£1718 to £2869)	Gamma: α = 7.4, β = 320.3	£2203 (£1566 to £2724)	Gamma: α = 6.3, β = 350.1
Cost of GAP implementation (software license and recurrent staff training) per birth	£10.80 (£10.36 to £11.20)	Gamma: α = 305.9, β = 0.03	£10.80 (£10.36 to £11.20)	Gamma: α = 305.9, β = 0.03
Birth outcome*				
% all births confirmed as SGA at birth	7.4 (6.3–8.3)	Beta: α = 22.0, β = 274.6	10.0 (8.3–11.7)	Beta: α = 14.1, β = 125.2
% undetected SGA neonates stillborn	0.98 (0.49–1.32)	Beta: α = 2.0, β = 206.8	0.81 (0.41–1.1)	Beta: α = 2.2, β = 272.3

Data are given as mean (interquartile range) [95% CI]. 95% confidence limits approximated as 2.5^th^ and 97.5^th^ percentiles from each output distribution. Primary SGA definition was birth weight < 10^th^ percentile according to both customized and population growth charts. Secondary SGA definition was birth weight < 10^th^ percentile according to customized growth chart for the Growth Assessment Protocol (GAP) and population charts for standard care.

* Variables not stratified according to intervention arm, as they were expected to vary only by screening outcome (for which GAP effect is already accounted) and not by intervention.

† Antenatal cost per birth under GAP was derived indirectly by adding the incremental cost of GAP (its treatment effect) to the antenatal cost per birth under standard care. FN, false negative; FP, false positive; NA, not applicable; TN, true negative; TP, true positive.

**Table 2:** Modeling output according to whether primary or secondary definition of small‐for‐gestational‐age (SGA) neonate was used: expected screening outcome

Model output parameter	*n* per 1000 births			
	Primary SGA definition		Secondary SGA definition	
	GAP	Standard care	GAP	Standard care
True positive	23	21	31	28
False negative	51	53	70	72
True negative	905	911	878	885
False positive	21	15	21	14

Primary SGA definition was birth weight < 10^th^ percentile according to both customized and population growth charts. Secondary SGA definition was birth weight < 10^th^ percentile according to customized growth chart for the Growth Assessment Protocol (GAP) and population charts for standard care.

**Table 3:** Modeling output according to whether primary or secondary definition of small‐for‐gestational‐age (SGA) neonate was used: incremental cost

Model output parameter	Primary SGA definition		Secondary SGA definition	
	Expected incremental cost of GAP (per 1000 births)	Probability that GAP increases cost (%)*	Expected incremental cost of GAP (per 1000 births)	Probability that GAP increases cost (%)*
GAP implementation cost (annual software license and recurrent staff training)	£10 796	100	£10 796	100
Incremental hospital care costs	£23 763	62	£20 065	60
Antenatal	£4754	54	–£21	50
Labor	£1122	60	£1308	59
Postnatal	£1721	61	£1966	61
Neonatal	£16 165	65	£16 812	66
Total incremental cost (implementation + hospital care)	£34 559	68	£30 861	65

Primary SGA definition was birth weight < 10^th^ percentile according to both customized and population growth charts. Secondary SGA definition was birth weight < 10^th^ percentile according to customized growth chart for the Growth Assessment Protocol (GAP) and population charts for standard care.

* Probability derived from proportion of model simulations resulting in positive incremental cost or clinical effect.

**Figure 2:**
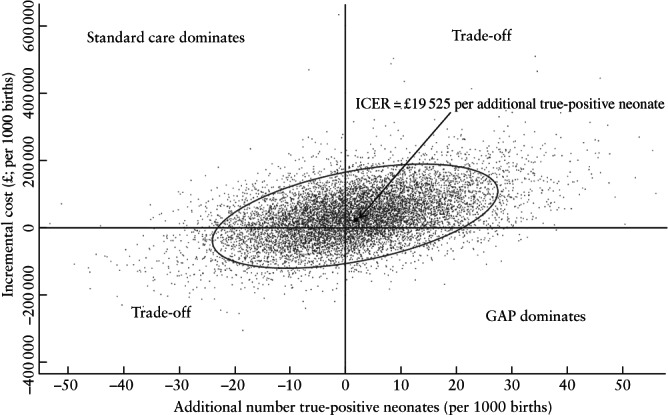
Cost‐effectiveness plane demonstrating the differences in costs and detection rate of small‐for‐gestational‐age neonate between standard care and Growth Assessment Protocol (GAP). The proportion of 10 000 incremental paired cost and clinical‐effect differences in each quadrant determines the probability of observing each of the four possible outcomes. Confidence ellipse is shown. ICER, incremental cost‐effectiveness ratio.

The expected clinical benefit of GAP in terms of antenatal detection of SGA, as observed in the DESiGN trial, was marginal. An additional 1.77 SGA cases were detected per 1000 births (55% probability that GAP would increase antenatal detection compared with standard care). The ICER for GAP using the primary SGA definition was £19 525 per additional SGA neonate identified, with a 44% probability that GAP would increase the total cost of care and be clinically beneficial compared with standard care. There was only an 11% probability that GAP would be superior to standard practice in terms of cost‐effectiveness, a 24% probability that standard care would be dominant and a 21% probability that GAP would reduce the total cost but would detect fewer SGA cases antenatally (Figure 2).

### Sensitivity analysis

The use of the secondary definition of SGA (SGA defined by customized centiles in GAP implementing clusters and by population centiles in standard‐care clusters) led to a reduction in the total incremental cost of GAP, to £30 861 per 1000 births, and a small increase in the expected number of additional SGA babies identified antenatally, to 2.52 per 1000 births. The ICER for GAP using the secondary SGA definition was £12 246 per additional case identified. Probability values were close to those observed in the primary analysis, with a 45% probability that GAP would increase the total cost of care but would be clinically beneficial, a 10% probability that GAP would be dominant, a 20% probability that standard care would be dominant and a 25% probability that GAP would reduce the total cost but would detect fewer SGA cases antenatally.

An uplift applied separately for antenatal appointments and unscheduled attendance costs had little impact on the total incremental cost of GAP (Appendix S4).

### Secondary analysis

**Figure 3:**
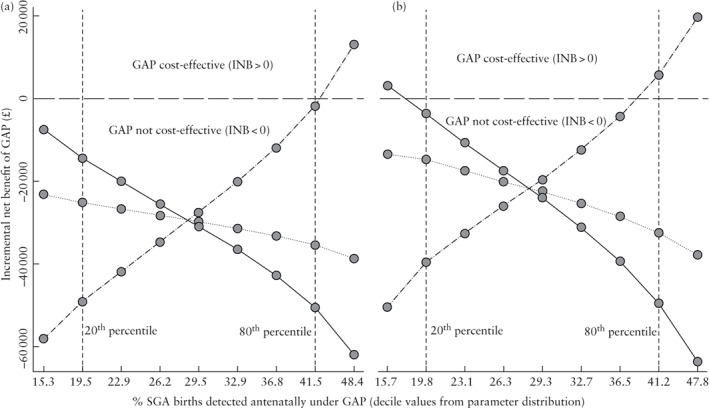
Expected incremental net benefit (INB) according to detection rate of small‐for‐gestational‐age (SGA) neonate by the Growth Assessment Protocol (GAP), based on primary (a) and secondary (b) definitions of SGA. Deciles from the expected distribution of SGA detection rate are represented by circles. Cost‐effectiveness, defined by INB, was determined using the UK National Institute for Health and Care Excellence cost‐effectiveness threshold, based on low (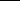), central (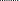) and high (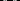) estimates of quality‐adjusted life years (QALYs) gained. INB > 0 implies that the gains in QALY from improved SGA detection outweigh the additional cost associated with GAP (i.e. GAP is cost‐effective compared with standard care), while INB < 0 implies that GAP offers poor value for money compared with standard care. The graph illustrates the rate of SGA detection that GAP would need to achieve to be a cost‐effective alternative to standard care and the statistical likelihood of such a rate. Overall, it suggests that the rate of SGA detection required to generate a cost‐effective outcome for GAP (INB > 0) is unlikely.

The incremental cost of GAP per additional infant QALY gained was estimated to exceed the NICE cost per QALY threshold (Table 4). QALY‐based ICERs ranged from £68 242 to £545 940 per QALY gained, depending on the assumptions adopted. Using the primary definition of SGA, GAP achieved a cost‐effective rate of antenatal detection only when adopting the high‐level assumptions regarding the QALY value of antenatal detection and only when the rate of detection exceeded 41.5% under GAP, which was on the 80^th^ percentile within the distribution for this parameter (Figure 3). Similar findings were observed for the analysis based on the secondary definition of SGA.

**Table 4:** Cost‐effectiveness of Growth Assessment Protocol (GAP) in terms of quality‐adjusted life year (QALY) gains arising from stillbirth prevention, judged against the NICE cost‐effectiveness threshold (CET), according to whether primary or secondary definition of small‐for‐gestational‐age (SGA) neonate was used

Estimate level	Primary SGA definition					Secondary SGA definition				
	Expected stillbirths avoided due to GAP (*n* per 1000 births)	Infant QALY gains per 1000 births	Expected cost per QALY gained (ICER)	Probability (%) that GAP is cost‐effective		Expected stillbirths avoided due to GAP (*n* per 1000 births)	Infant QALY gains per 1000 births	Expected cost per QALY gained (ICER)	Probability (%) that GAP is cost‐effective	
				CET = £20K per QALY gained	CET = £30K per QALY gained				CET = £20K per QALY gained	CET = £30K per QALY gained
High estimate	0.017	0.51	£68 242	38	41	0.019	0.58	£52 993	40	43
Central estimate	0.008	0.20	£172 547	35	35	0.010	0.23	£133 991	36	37
Low estimate	0.004	0.06	£545 940	33	33	0.005	0.07	£423 948	35	35

Central estimate assumed that 50% of stillbirths linked to undetected SGA are prevented with 23.73 QALYs gained per stillbirth avoided; high estimate assumed all stillbirths prevented, with 30 QALYs gained; low estimate assumed 25% of stillbirths prevented, with 15 QALYs gained. Probability based on proportion of incremental net benefit values across model simulations that are > 0 (i.e. where GAP is cost‐effective). ICER, incremental cost‐effectiveness ratio.

## DISCUSSION

### Main findings

This cost‐effectiveness analysis, based on data from sites recruited into the DESiGN trial, suggests that the adoption of GAP in place of standard care would (on average) increase costs to NHS providers while offering only marginal clinical benefit. After full consideration of the margins of uncertainty around economic and clinical parameters of relevance, the expected incremental cost of GAP was estimated to be £34 559 per 1000 births, although some uncertainty remains regarding the magnitude of this effect (68% probability that the incremental cost of GAP would be positive and 32% probability that GAP would reduce the costs). Overall, 31% of the expected incremental cost of GAP was attributable to implementation of the program. Compared with other estimated resource effects, these costs are least affected by sampling uncertainty inherent to a clinical trial. There was no convincing evidence that GAP was the dominant clinical strategy in terms of cost‐effectiveness (11% probability that it would reduce costs while also increasing the rate of SGA detection). Taking into account sampling error in the trial data, GAP allowed only marginal expected clinical gains, at an additional cost, at the NHS sites that participated in the trial. A secondary analysis revealed no convincing evidence that GAP provided a cost‐effective alternative to standard care within participating clusters when applying to our findings a cost‐effectiveness threshold routinely adopted by the NHS.

### Strengths and limitations

The main strength of our study is that the evaluation was based on resource‐use data recorded routinely during clinical practice. It is therefore likely to offer a reliable assessment of the impact of GAP implementation on hospital resources within the study sites randomized to the program. GAP was compared with current standard practice, rather than with absence of care. Therefore, the findings reflect the expected increase in cost associated with implementing GAP compared with current practice (analysis method recommended by NICE)^29^.

One limitation of our study is the adopted time horizon. Estimates of cost were restricted to those incurred by the NHS provider until the end of the care episode that included birth. We did not account for infant and adult healthcare costs due to morbidity associated with preterm or early term birth that may follow SGA detection or intermediate and long‐term health or social costs associated with prevention of stillbirth, including any costs of litigation (estimates have been published previously)^30^.

We were also limited by the availability and quality of data collected from some clusters. One site implementing GAP was excluded from some analyses because it could not provide data on true‐negative and false‐positive SGA diagnoses. Hospital administrative data were missing entirely or could not be used for two sites allocated to standard care and were missing partially (but systematically) for some resource items at three of the remaining standard‐care clusters. Exclusion of scheduled and unscheduled antenatal hospital appointments or day attendances because of these missing data had only a small effect on ICER, as shown by the sensitivity analysis. In all clusters, we were unable to distinguish between women who had absence of an activity recorded because it had occurred elsewhere, those who had not undergone the activity anywhere and those who underwent the activity, but without it being recorded. We introduced assumptions to which plausible limits were applied to deal with this issue.

Another limitation is the choice of primary SGA definition based on both population and customized weight charts. Using this definition, false‐positive screening outcomes were defined in approximately 5% of babies^5^ that met criteria for SGA at birth according to either the customized or the population chart, but not according to both chart types. A sensitivity analysis that adopted an SGA definition that would be more likely to be applied in routine clinical settings (SGA defined by customized centiles in GAP clusters and by population centiles in standard‐care clusters) produced a lower ICER (£12 246 per SGA baby detected correctly) with comparable estimates of uncertainty around observing alternative cost‐effectiveness outcomes. As also demonstrated in the analysis using the primary SGA definition, application of the secondary SGA definition showed that GAP would have delivered a cost‐effective outcome when judged against a routinely applied cost‐effectiveness threshold used in NHS decision‐making only if an unlikely combination of assumptions was built into the analysis.

Finally, the findings and implications of this economic evaluation are applicable only to healthcare systems that have similar resource availability and national protocols to those of the clusters included within the DESiGN trial.

### Interpretation

The cost‐effectiveness of GAP has not been studied previously. The GAP provider conducted a cost‐benefit analysis in which the effect of increasing the frequency of fetal growth scans for women at high risk of SGA was studied^31^. Based on estimates regarding the relationship between SGA detection and infant outcome (prevention of one stillbirth per 1000 births, £20 000 per 1000 births saved due to reduced neonatal admissions, £25 000 per 1000 births saved due to reduction in the rate of cerebral palsy and £70 000 per 1000 births saved due to reduced litigation rate), this analysis predicted cost savings of £120 000 per 1000 births, which was attributed to fewer neonatal admissions and lower rates of perinatal morbidity, mortality, cerebral palsy and litigation. Our analysis differed in scope, as we did not consider costs of litigation or long‐term outcomes and drew on data generated from a ‘gold‐standard’ research design linked directly to the implementation of GAP within NHS maternity settings. These differences between their analysis and ours may explain the discrepancies in findings. Whilst we acknowledge that improved SGA detection is expected to reduce both stillbirth and long‐term disability related to fetal brain injury, the DESiGN trial found only marginal differences in rates of SGA detection with GAP implementation, and these differences were not statistically significant.

Our economic evaluation is not supportive of GAP providing a cost‐effective improvement to care processes aimed at prevention of stillbirth. The expectation based on evidence from this evaluation is that it will increase the costs of hospital care and require an ongoing resource commitment in terms of staff training and software licensing. These additional costs need to be balanced against the small expected incremental clinical benefit that GAP may offer compared with standard care. We estimated that, even with highly optimistic (and arguably unrealistic) assumptions regarding preventable numbers of stillborn infants arising from early detection, the QALY value of stillbirth prevention linked to these small clinical gains would be of insufficient magnitude to justify the costs when judged against cost‐effectiveness thresholds used in NHS decision‐making. It is likely that this conclusion would have been strengthened if our analysis had included long‐term NHS costs and impact on QALYs arising from stillbirth prevention and iatrogenic preterm birth. Other long‐term cost‐ and QALY‐related benefits that it has been claimed are linked to the early detection of SGA birth (e.g. prevention of litigation costs, perinatal morbidity and long‐term developmental disorders) would need to be substantial to offset our core findings. This seems unlikely, given the low additional rates of antenatal detection observed using GAP in this study.

### Conclusion

The economic case for replacing standard care with GAP is weak based on the analysis and evidence reported herein. This conclusion should be viewed taking into account that cost‐effectiveness analyses are always limited by the assumptions made, and our study is no different.

## Supporting information


**Appendix S1** Additional description of study methodology

**Appendix S2** Activities within maternity and neonatal care pathways that were hypothesized to vary with implementation of the Growth Assessment Protocol (GAP)

**Appendix S3** Cost inputs for economic model

**Appendix S4** Results of sensitivity analysis

## Data Availability

The data that support the findings of this study are available on request from the corresponding author. The data are not publicly available due to privacy or ethical restrictions.
