# Supplementary material for: Improving antenatal detection of small‐for‐gestational‐age fetus: economic evaluation of Growth Assessment Protocol
Source: Ultrasound Obstet Gynecol. 2022 Nov 1;60(5):620–31. doi: 10.1002/uog.26022 (PMC9828078; doi:10.1002/uog.26022)
Supplement: Supplementary file 1 — Appendix S1 Additional description of study methodology [file UOG-60-620-s004.pdf]

Appendix 1 – Additional Description of Methods

Clinical Outcome

Fetal SGA was defined as estimated weight <10th centile for gestational age and sex by a population reference chart (Hadlock fetal charts) in the standard care arm and by customised GROW charts in clusters allocated to GAP. To ensure that the outcomes were comparable across both trial arms, the denominator (SGA at birth) was defined as a birthweight which met the definition for SGA (i.e. <10th weight centile) by both the population reference (UK 1990 birthweight charts) and the customised standard (GROW) charts.

Unit costs

For each item of activity, the most recent and nationally-appliable cost was identified, which was sufficiently granular to cost the single activity item (rather than a bundled cost for a composite of activity items). Cost sources were prioritised in the following order: (1) National reference costs, (2) economic evaluations nested within national guidelines, (3) economic evaluations nested within national Health Technology Appraisals, (4) costs derived from published economic evaluations nested within research trials or observational studies.

Costs were then inflated where appropriate to 2018-19 prices, using the Department of Health's Pay & Price Series for financial years 2008/09 - 2015/16 and the NHS Improvement Economic Assumptions for years 2016/17 to 2018/19. [1 2]

All midwifery staff were assumed to be band 6 on the pay scale, all sonographers were assumed to be band 7, the average registrar salary was used for junior doctors.

Management of missing data

For hospital activity data, we treated the following as missing and predicted the missing values with multiple imputation using chained equations: number of ultrasound scans after 24

weeks' gestation if the woman had no record of fetal screening scans prior to 24 weeks', number of antenatal clinic appointments or length of antenatal admission after 24 weeks' gestation if the woman had no record of appointments prior to 32 weeks', postnatal length of stay if no data available. Data were imputed within cluster and therefore only imputed if not completely missing at the originating cluster site. Neonates without a record of admission to a neonatal care unit were assumed to have not been admitted. Where a site did not contribute any data on a resource item, it was excluded from the analysis.

Modelling parameter estimation: cluster summary approach

The economic evaluation utilised data from the DESiGN study – a randomised cluster control trial that compared the effect of GAP and standard care. Analysis of birth/pregnancy-level data would therefore risk underestimating variances and misrepresenting second-order uncertainty around the values of important input parameters for the economic modelling. We adopted a cluster summary approach to deal with this. This method is specifically recommended where the number of clusters is small,[3-5] preventing use of more detailed multilevel modelling to deal with the effects of clustering (in the DESiGN study only 13 hospitals randomised).

The model contains 5 sets of input parameters: the proportion of 1000 births expected to be identified as either SGA or not SGA (P0 and 1-P0 in figure 1); the proportion of SGA births defined as "true positive" or false negative" (the screening outcome) depending on whether SGA was detected antenatally (P1 and 1-P1); the proportion of non-SGA births defined as "false positive" or "true negative" (P2 and 1-P2); the cost of clinical resource use per birth for each stage of care by screening outcome; and the cost per birth of associated with implementing the GAP programme. This model structure captures the way that GAP is expected to impact on cost within the care pathway: by increasing the proportion of true positive SGA births and by potentially reducing the proportion non-SGA births that are false positive (P1 and P2); and by increasing the costs of antenatal care per birth above what would have been expected had standard practice

been applied to any pregnancy detected as SGA antenatally (principally through increasing the frequency of scans performed).

Screening outcome proportions and mean cost by screening cluster summaries were derived using hospital-specific predicted values derived from multivariate statistical models fitted to pregnancy-level data from the clinical trial. Logit models were estimated separately for SGA and non-SGA births using a binary dependent variable for clinical outcome (true positive or false positive=1). Both models were used to predict the proportion of births expected for clinical outcome at each hospital. For cluster summary costs, separate models were estimated using antenatal, intrapartum, postnatal, and neonatal costs as the dependent variable. All cost models used generalised linear modelling (GLM)[6] applying a log-link function. Choice of distributional family was assessed using a modified Park test (ref).[6 7] Negative binomial regression was implemented instead of a Poisson model (where indicated by the Park test) if overdispersion was evident. Hospital fixed effects, maternal ethnicity, parity, age, and body mass index (BMI; cost modelling only) were included as covariates in all models. Adjustment for the pregnancy risk covariates served a dual purpose: to obtain cluster summary estimates of cost across the 4 clinical outcomes that reflected incremental clinical resource utilisation in response to positive case identification (whether true or false positive) over and above any independent effect of pregnancy risk factors; and to adjust for hospital "case-mix" when building-in any treatment effect into the economic modelling associated with GAP on screening outcome and antenatal cost (see below). Screening outcome was also added as a covariate to each cost model to facilitate predictions by clinical outcome. For antenatal costs there was an expectation that any differences in cost between GAP and standard care clusters would principally be concentrated in those pregnancies where SGA was detected antenatally. Interaction effects between screening outcome and site clusters were therefore built into the antenatal cost model to so that the magnitude of between-site differences in cluster summary cost values would be allowed to differ according to screening outcome.

Treatment effects

The economic modelling underpinning the cost-effectiveness analysis builds in any effect of GAP on antenatal resource use and the likelihood of observing alternative screening outcomes. For antenatal costs this involved fitting a linear regression model to the cluster summary values for antenatal cost for each screening outcome, including a binary treatment allocation identifier for each site cluster as a covariate. Treatment effect on clinical outcome similarly involved fitting a linear regression model to a logit transformation of the cluster summary outcome proportions to ensure that re-transformed values used for economic modelling were constrained to lie between zero and one.[8]

To reflect uncertainty in the input parameters arising from trial sampling errors, a distribution of parameter values was generated through repeated random sampling from a probability distribution determined for each parameter using the trial data. The variance-covariance matrices from both models, combined with the linear predictors, were used to promulgate uncertainty around differences in outcome values into the economic modelling using the Cholsesky decomposition method referred to in the main text. Both regression models controlled for: a baseline case-mix adjusted site cluster prediction of antenatal cost/screening outcome proportions; and a variable adjusting for randomisation strata. The baseline predictions were obtained by fitting the models described in the previous section to pregnancy-level trial data obtained for a 12-month pre-implementation baseline period. The values from these distributions are then combined with reference to the structure of the model shown in Figure 1 to create a distribution of values for the total cost of care and the number of true positive births under GAP and standard care. Inferences regarding the cost-effectiveness of GAP are made with reference to the expected (or the mean) value from both these output distributions.

Sensitivity analysis

To assess whether exclusion of triage contacts and antenatal appointments from antenatal costs may have biased comparisons between GAP and standard care we repeated our

probabilistic analysis of total costs of care under alternative scenarios: exclusion of both these items (the base case analysis); the base case plus an uplift for triage costs; and the base case plus an uplift for appointment costs. Probability distributions for antenatal costs based on cluster summary values consistent with the base case definition of antenatal cost (antenatal admissions and scans only) and then with the separate inclusion of the two additional items were generated (using only site clusters with available data for all items). The ratio between the "new" and the base case values were used as uplift factors and used to generate alternative values for the total cost of care within the model cohort.

References

1. Department of Health. Pay & Price Series 2015/16 2016 [Available from: www.info.doh.gov.uk accessed 11 December 2018.
2. NHS Improvement. Economic Assumptions for 2016/17 to 2020/21. 2016 [Available from: https://www.gov.uk/government/publications/economic-assumptions-201617-to-202021/economic-assumptions-201617-to-202021 accessed 11 December 2018.
3. Hayes R, Moulton L. Cluster Randomized Trials. Abingdon, UK: Taylor & Francis 2009.
4. Campbell MK, Mollison J, Steen N, et al. Analysis of cluster randomized trials in primary care: a practical approach. *Fam Pract* 2000;17(2):192-6. doi: 10.1093/fampra/17.2.192 [published Online First: 2000/04/12]
5. Zucker DM, Lakatos E, Webber LS, et al. Statistical design of the Child and Adolescent Trial for Cardiovascular Health (CATCH): implications of cluster randomization. *Control Clin Trials* 1995;16(2):96-118. doi: 10.1016/0197-2456(94)00026-y [published Online First: 1995/04/01]
6. Glick H, Doshi J, Sonnad S, et al. Economic Evaluation in Clinical Trials. Oxford, UK: Oxford University Press 2015.
7. Manning W, Mullahy J. Estimating log models: to transform or not to transform? *Journal of Health Economics* 2001;20(4)
8. Baum C. Modelling proportions. . *Stata J* 2008;8(2):299-303.
