# Supplementary material for: Improving antenatal detection of small‐for‐gestational‐age fetus: economic evaluation of Growth Assessment Protocol
Source: Ultrasound Obstet Gynecol. 2022 Nov 1;60(5):620–31. doi: 10.1002/uog.26022 (PMC9828078; doi:10.1002/uog.26022)
Supplement: Supplementary file 2 — Appendix S2 Activities within maternity and neonatal care pathways that were hypothesized to vary with implementation of the Growth Assessment Protocol (GAP) [file UOG-60-620-s002.pdf]

**Activities within the maternity and neonatal care pathway which were hypothesised to potentially vary with implementation of GAP**

| **Antenatal care** (after 24 weeks' gestation) | **Intrapartum care** | **Postnatal care** | **Neonatal care** |
|---|---|---|---|
| • Antenatal appointments<br>• Attendance to day assessment unit/triage<br>• Antenatal inpatient admission<br>• Ultrasound scan for fetal growth<br>• Ultrasound scan for fetal growth | • Induction of labour<br>• Epidural<br>• Unassisted vaginal birth<br>• Assisted vaginal birth<br>• Elective Caesarean section<br>• Emergency Caesarean section<br>• Repair $3^{rd}/4^{th}$ degree tear<br>• Treatment of postpartum haemorrhage (500mL-1500mL)<br>• Treatment of major obstetric haemorrhage (>1500mL) | • Maternal stay in postnatal ward (with/without baby) | • Admission to neonatal intensive care unit<br>• Admission to neonatal high dependency unit<br>• Admission to special care baby unit<br>• Neonatal transitional care |
