# Supplementary material for: Improving antenatal detection of small‐for‐gestational‐age fetus: economic evaluation of Growth Assessment Protocol
Source: Ultrasound Obstet Gynecol. 2022 Nov 1;60(5):620–31. doi: 10.1002/uog.26022 (PMC9828078; doi:10.1002/uog.26022)
Supplement: Supplementary file 3 — Appendix S3 Cost inputs for economic model [file UOG-60-620-s001.pdf]

Cost inputs for the economic model

Annual cost of implementing GAP (2018/19)

Annual birth rate	Set up cost (includes whole day of training)	Annual cost
<3000 babies	£500	£1500
3000-5000 babies	£500	£2000
5000 – 7000 babies	£500	£3000
>7000 babies	£500	£4000

Salary costs used in economic evaluation

Cost type	Midwife (band 6)	Sonographer (band 7)	Consultant obstetrician	Junior doctor (Registrar)
**Annual salary**	£32,563	£39,181	£90,535	£41,583
**Annual on-costs**	£8,050	£9,912	£24,386	£10,591
**Other overheads**	£30,794	£42,638	£83,457	£39,823
**Total annual cost**	**£71,407**	**£91,731**	**£198,378**	**£91,897**
**Annual hours of work (full time)**	1,573	1,599	1,842	2,138
**Estimated hourly cost**	**£45**	**£57**	**£108**	**£43**

Clinical care activities and maternal or neonatal outcomes and costs estimated for the cost-effectiveness analysis

Cost item	Cost applied (inflated to 2018/19)	Cost source
**Antenatal costs**		
Standard midwifery outpatient appointment	£117.16 / appt	NHS reference costs 2015-16[269]
Standard obstetric outpatient appointment	£141.95 / appt	
Standard outpatient appointment (lead professional not known)	£129.56 / appt	Midpoint between midwife and consultant costs (above).
Attendance to maternity day assessment unit or maternity triage centre.	£259.48 / attendance	NHS reference costs 2015-16 (NZ16Z - Day case)[269]
Admission on antenatal ward	£1,133.76 for first day and £184.67 for every day which follows	NHS reference costs 2015-16 (NZ16Z - Antenatal Routine Observation)[269]
Fetal growth ultrasound scan*	£109.07 / scan	NHS reference costs 2015-16 (NZ21Z)[269]
**Intrapartum costs**		
Induction of labour (includes admission)	£394.71	NICE – Inducing Labour (2008)[375]
Epidural	£118.08	NICE – Intrapartum Care for healthy women and babies (2014)[338]
Spontaneous vaginal birth	£1,762.19	
Instrumental vaginal birth	£2,663.45	
Elective Caesarean Section	£3,923,25	
Emergency Caesarean Section	£3,923.25	
Repair 3/4th degree tear	£351.95	
Postpartum haemorrhage (500-1500mL)	£100.32	
Postpartum haemorrhage > 1500mL	£1,140.99	
**Postnatal costs**		
Inpatient stay on postnatal ward	£431.56 / day	NICE – Intrapartum Care for healthy women and babies (2014)[338]
**Neonatal costs**		
Admission to Neonatal Intensive Care Unit (level 3)	£1,157.70 / day	NHS reference costs 2017-18 XA01Z[293]
Admission to Local Neonatal Unit (level 2)	£780.30 / day	NHS reference costs 2017-18 XA02Z[293]
Admission to Special Care Baby Unit (level 1)	£542.64 / day	NHS reference costs 2017-18 XA03Z[293]

*Data which indicated which healthcare professional conducted the scan were not available at most sites and so all scans are assumed to have been sonographer-led.
