# Supplementary material for: Improving antenatal detection of small‐for‐gestational‐age fetus: economic evaluation of Growth Assessment Protocol
Source: Ultrasound Obstet Gynecol. 2022 Nov 1;60(5):620–31. doi: 10.1002/uog.26022 (PMC9828078; doi:10.1002/uog.26022)
Supplement: Supplementary file 4 — Appendix S4 Results of sensitivity analysis [file UOG-60-620-s003.pdf]

Results of sensitivity analysis

Cost-effectiveness analysis: With uplift for antenatal appointment cost[*]

**Incremental cost of GAP (per 1000 pregnancies)**	**Expected value**	Probability that GAP is cost increasing
**Antenatal cost**	£5,263	54%
**Total incremental cost**	£35,069	67%
**Incremental cost per additional true positive neonate (ICER)**	£19,813	
**Probability (%) that GAP increases total cost of care and detects more SGA neonates antenatally**	43.98%	
Probability GAP is dominant	11.42%	
Probability standard care is dominant	23.15%	
Probability GAP reduces total cost and detects fewer SGA neonates antenatally	21.45%	

1. Model outputs unaffected by uplift are not reported

*Cost-effectiveness analysis: With uplift for antenatal triage cost**

**Incremental cost of GAP (per 1000 pregnancies)**	**Expected value**	Probability that GAP is cost increasing
**Antenatal cost**	£6,013	54%
**Total incremental cost**	£35,818	67%
**Incremental cost per additional true positive neonate (ICER)**	£20,236	
Probability (%) that GAP increases total cost of care and detects more SGA neonates antenatally	44.47%	
Probability GAP is dominant	10.93%	
Probability standard care is dominant	22.96%	
Probability GAP reduces total cost and detects fewer SGA neonates antenatally	21.64%	

*Model outputs unaffected by uplift are not reported
